# Remote Sensory–Cognitive Assessment in Children with Autism: Evaluating Feasibility and Performance Outcomes

**DOI:** 10.3390/bs16050702

**Published:** 2026-05-04

**Authors:** Peige Wang, Lakshmi Kannan, Ellie Hodge, Jennie Marie Sanchez-Singh, Audrey Anna Carrillo, Sophia Milla, Katherine Meltzoff, Aaron R. Seitz

**Affiliations:** 1 Institution for Cognitive and Brain Health, Department of Psychology, Northeastern University, 360 Huntington Ave, Boston, MA 02115, USA; wang.peig@northeastern.edu (P.W.); kannanl@ohsu.edu (L.K.); milla.s@northeastern.edu (S.M.); 2 Social Cognitive Developmental Neuroscience Lab, School of Education, University of California, 900 University Ave, Riverside, CA 92521, USA; ellie.hodge@ucr.edu; 3 Brain Game Center for Mental Fitness and Well-Being, Northeastern University, 1201 University Avenue #204, Riverside, CA 92521, USA; jenniemsanchez@gmail.com (J.M.S.-S.); audrey.carrillo@ucr.edu (A.A.C.)

**Keywords:** autism spectrum disorder, remote assessment, sensory processing, data quality

## Abstract

This study evaluated the feasibility of remotely administered, multi-session digital sensory–cognitive assessments in children with autism spectrum disorder (ASD). Remote assessment in ASD presents challenges, including sustaining engagement across sessions, ensuring task comprehension, and maintaining data quality in home environments. Accordingly, this study quantified feasibility through task engagement and data integrity across a multi-domain assessment battery. A total of 121 children with ASD aged 8–16 years participated. The assessment battery was administered across three remote sessions targeting emotion discrimination, visual and cognitive processing, and auditory processing. Feasibility was evaluated using a structured data-quality coding system integrating trial-level performance with qualitative observations of engagement and data integrity. Across 11 behavioral tasks, an average of 85% of participants produced usable data. Exclusions occurred across most tasks but were broadly distributed, with no single task showing disproportionately elevated failure rates. Among participants with usable data, performance distributions fell within expected ranges. These findings indicate that remote sensory–cognitive assessment is feasible in children with ASD, supporting the scalability of multi-domain digital assessments and providing a foundation for larger studies investigating individual variability across perceptual and cognitive domains.

## 1. Introduction

Autism Spectrum Disorder (ASD) is a neurodevelopmental condition diagnosed on the basis of two overarching behavioral characteristics: challenges with social interactions, and restricted, repetitive patterns of behavior, interests, or activities (Regier et al., 2013). Importantly, heterogeneity extends beyond diagnostic features where individuals with ASD show distinct patterns of strengths and weaknesses across multiple domains (Halder et al., 2024; Mottron & Bzdok, 2020; Uljarević et al., 2017). These domains include low-level perceptual processes (Uljarević et al., 2017), such as visual and auditory signal detection and discrimination (Chung & Son, 2020; Ong et al., 2024); higher-order domains, including filtering stimuli (Joseph et al., 2009; Wasiuk et al., 2025), integrating information (Bertone et al., 2003; Harms et al., 2010; Marco et al., 2011), working memory (Habib et al., 2019); and other executive function domains (Demetriou et al., 2019). As a result, such variability contributes to the contradictory findings in studies of perception (Kaldy et al., 2016; Simmons et al., 2009), emotion recognition (Harms et al., 2010), and executive function (St John et al., 2022). Together, this body of evidence highlights ASD as a multidimensional condition in which domain-specific cognitive differences may contribute to diverse behavioral outcomes (Happé et al., 2006). Comprehensive assessments across perceptual and cognitive domains are therefore critical for characterizing individual variability and advancing our understanding of ASD heterogeneity.

Despite growing recognition that comprehensive sensory–cognitive assessments is essential for all individuals with ASD (Lord et al., 2018), scalable and widely accessible implementations remain rare in practice. Traditional studies often rely on geographically restricted samples (Masoomi et al., 2025) and narrowly focused tasks (Kaldy et al., 2016; Remington & Fairnie, 2017), constraining both generalizability and the ability to capture the breadth of sensory and cognitive variability observed in ASD (Lenroot & Yeung, 2013; Lombardo et al., 2019). Remote testing offers a promising alternative by reducing barriers related to geographic access, scheduling constraints, and anxiety associated with laboratory or clinical settings (Katakis et al., 2025). However, remote assessment in autistic populations is frequently accompanied by high attrition rates (Whitehouse et al., 2017), raising concerns about the feasibility of unsupervised, at-home assessments (Germine et al., 2012). A key challenge is the absence of real-time scaffolding from trained researchers, which can hinder task comprehension and sustained engagement, particularly for individuals with attentional or sensory processing differences (Calub et al., 2022; Panesi et al., 2023). Additional factors such as environmental distractions (e.g., noise, lighting), inconsistent caregiver involvement (e.g., under- or over-assistance), and technical variability (e.g., device type, screen size, or audio fidelity) further threaten data usability and interpretability in remote contexts (Reips, 2002).

**Figure 1:**
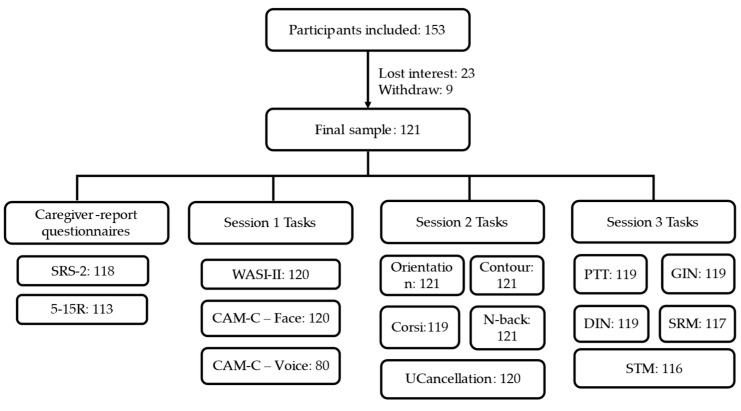
Flow Diagram of Participant Retention Across Remote Assessment Sessions. Arrows indicate the progression of participants across sessions.

To address these feasibility barriers and make remote assessment more valid, scalable, and replicable for the ASD population, we designed a digital assessment battery across multiple domains implicated in ASD heterogeneity (Figure 1), including auditory and visual processing, cognitive control and working memory, and complex emotion recognition. Task and questionnaire selection was guided by the goal of capturing complementary aspects of sensory, cognitive, and behavioral functioning. Parent-report measures were included to provide standardized indices of social and developmental functioning, and to enable future investigation of links between perceptual performance and real-world outcomes. Visual and auditory tasks were chosen to probe central perceptual processing, with particular emphasis on lower- and mid-level mechanisms that have been relatively underexplored in ASD. Cognitive tasks targeted key executive functions, including working memory and inhibitory control, and were implemented in a gamified format to support engagement in a remote, child-friendly context. These tasks were implemented in a simple to use mobile application BGC Science (Brain Game Center, n.d.) that participants could download on their own devices (phones or tablets). In addition, trained research assistants (RA) maintained continuous communication with participants throughout the assessment process, providing live support when needed to ensure task comprehension and sustained engagement.

In the present feasibility study, we examined acceptability of the PART/BGC Science (Brain Game Center, n.d.) platform and its ability to collect usable data under remote administration. Feasibility was operationalized by examining patterns of task engagement and data usability across the assessment battery, with the goal of determining whether remote assessment could be implemented successfully and, if not, where feasibility breakdowns occurred. This early-stage work aims to set the ground for future studies that could use apps of these types to better characterize perceptual and cognitive profiles and how these may relate to higher-level social–emotional functioning.

## 2. Materials and Methods

### 2.1. Participant

**Figure 2:**
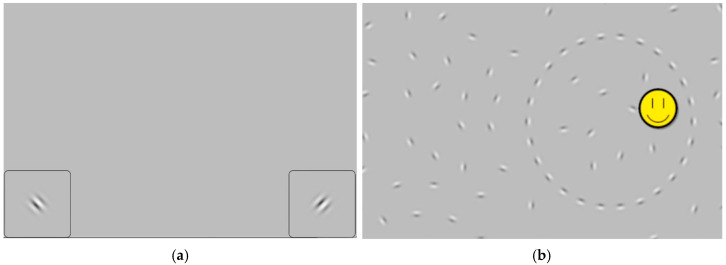

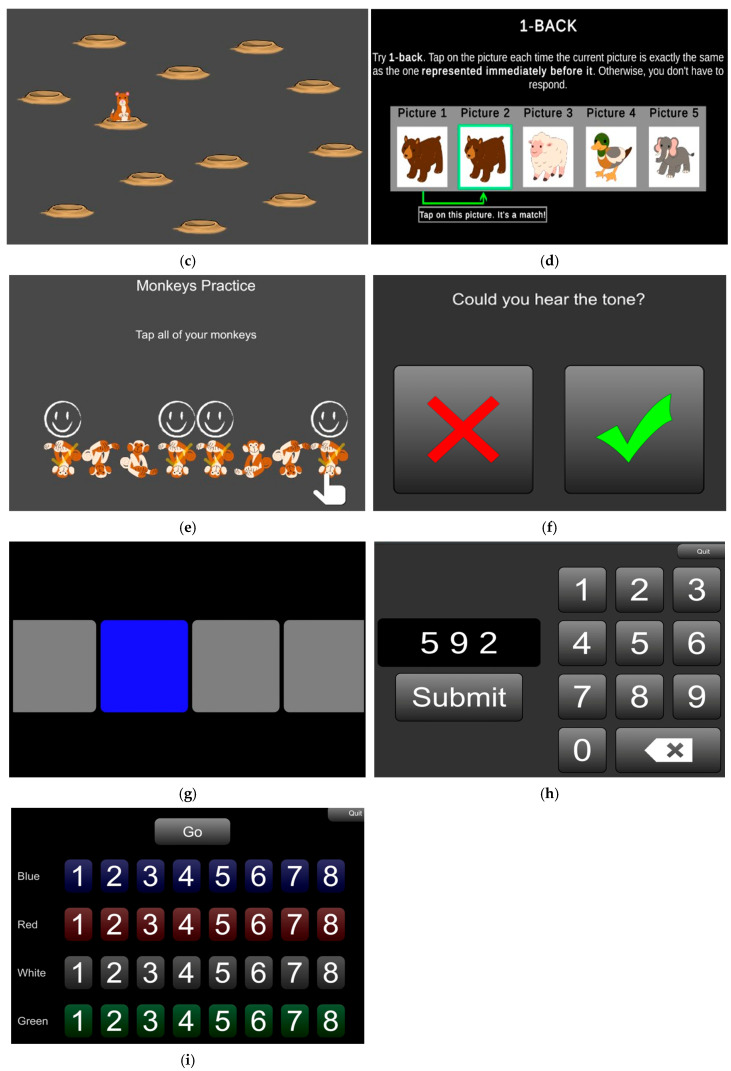
Representative Screenshots of the Administered Tasks. (**a**) Orientation Discrimination task; (**b**) Contour Integration task; (**c**) Corsi Task; (**d**) N-Back Task; (**e**) UCancellation Task; (**f**) Pure Tone Task; (**g**) Gap in Noise and Spectrotemporal Modulation Task; (**h**) Digits in Noise Task; (**i**) Spatial Release Masking Task.

Two hundred and four participants with a prior diagnosis of ASD, as reported by parents, were recruited through “SPARK for Autism”, “Children Helping Science”, or general lab outreach. Of the 204 participants screened, 153 met the inclusion criteria. Of these, 9 participants withdrew and 23 were lost to follow-up, resulting in a final analytic sample of 121 participants who completed at least part of the assessment protocol. Because the study was conducted across multiple remote sessions and tasks were introduced in a staggered manner, not all participants completed all assessments (see Figure 2). Demographic characteristics of the final analytic sample are reported in Table 1.

All procedures were approved by the University of California, Riverside Institutional Review Board (IRB-SB. NUMBER: #HS-20-177). Participants received a $10 per hour Amazon gift card as compensation for their time.

Inclusion criteria were designed to ensure both clinical appropriateness and feasibility of remote participation. Criteria included: (a) a parent-reported clinical diagnosis of ASD; (b) access to required technology for remote testing, including two internet-enabled devices (iOS or Android smartphone, tablet, or computer), headphones, at least 2.5 GB of available storage to download the BGC Science, an app made by Brain Game Center for Mental Fitness and Well-Being and distributed freely online, and an active email address for account activation; (c) basic English proficiency; (d) normal or corrected vision sufficient to see stimuli on a screen at a distance of approximately two feet; (e) no parent-reported diagnosis of any psychiatric disorder besides ASD (e.g., schizophrenia, bipolar disorder, ADHD, depression, anxiety), intellectual disability, or significant perinatal complications (i.e., birth weight under 4 lbs or gestational age under 36 weeks), (f) full-scale IQ of 70 or above. To ensure participants had a full-scale IQ of 70 or above, all participants completed the two-subtest version of Wechsler Abbreviated Scale of Intelligence–Second Edition (WASI-II).

### 2.2. Research Design

The study employed a supervised, remotely administered assessment design comprising three child testing sessions and parent-completed questionnaires. The three child sessions were structured to target distinct domains of sensory and cognitive function, whereas caregiver-report measures were completed independently by parents to provide contextual and symptom-level information.

Caregiver-report questionnaires (i.e., SRS-2, 5-15R) were completed online by parents outside of the child testing sessions.

Session 1 focused on emotion discrimination and general cognitive ability. Measures included Child Affective Measure—Child Version (CAM-C), which provided accuracy scores for facial and vocal emotion recognition, and the WASI-II, which yielded age-normed estimates of verbal and nonverbal intellectual functioning. Session 2 assessed visual perception and cognitive functioning. Visual tasks (orientation discrimination and contour integration) generated threshold estimates of fine-grained spatial sensitivity and global contour integration, respectively. Cognitive tasks (N-back, Corsi, and Cancellation) produced indices of working memory updating, visuospatial span, and inhibitory control. Session 3 evaluated auditory perception across multiple processing levels. Pure tone detection measured minimum audibility thresholds; gap-in-noise assessed temporal resolution; digits-in-noise and spatial release from masking indexed speech-in-competition ability; and spectrotemporal modulation tasks quantified sensitivity to dynamic spectral–temporal structure (see Figure 1).

Feasibility of remote assessment was evaluated across sessions using structured data quality coding.

### 2.3. Procedure

Participants completed three remote child testing sessions, each lasting approximately 30–60 min, with a minimum interval of 24 h between sessions. Prior to the first session, parents received an email with instructions for downloading and activating the BGC Science application.

Caregiver-report questionnaires were distributed electronically and completed asynchronously by parents via Qualtrics. These measures did not require live supervision and could be completed at the parents’ convenience.

All child sessions were conducted via Zoom and facilitated by a trained RA. Training for RAs included detailed instruction on task procedures, standardized administration protocols, and participant interaction guidelines, as well as supervised practice sessions to ensure consistency across sessions. During each session, the RA read aloud on-screen task instructions, clarified questions as needed, and repeated instructions when necessary to ensure task comprehension. Throughout testing, the RA monitored participant behavior and documented contextual, behavioral, and technical factors that could compromise data quality, including excessive background noise, technical issues (e.g., app malfunction or faulty headphones), and signs of inattention or impatience. Behavioral indicators included both verbal expressions (e.g., complaints or off-task conversation) and nonverbal cues (e.g., slouching or leaving the screen).

### 2.4. Measures

#### 2.4.1. Caregiver-Report Questionnaires

Social Responsiveness Scale–Second Edition (SRS-2). The SRS-2 is a 65-item questionnaire designed to assess social behavior impairments associated with ASD (Bruni, 2014). Items are rated on a 4-point Likert scale ranging from 1 (Not True) to 4 (Almost Always True). A total T-score was computed based on five domains: Social Awareness, Social Cognition, Social Communication, Social Motivation, and Restricted Interests and Repetitive Behavior were computed. Higher T-score indicated greater social-communicative difficulties, with T-scores of 76 or higher indicating severe social challenges, 66–75 indicating moderate, 60–65 suggesting mild difficulties, and 59 and below suggesting minimal to no impairment. The scale demonstrated strong psychometric properties, with an internal consistency (α = 0.95) in clinical samples (Bruni, 2014). There were 12 participants with SRS scores under 59 (i.e., scores do not indicate social-communicative challenges). A T-test for all questionnaires and tasks was run comparing participants with SRS scores above and below 59. Only the orientation discrimination task evidenced significant differences between groups. Therefore, participants with scores under 59 were included in the analyses.

Five to Fifteen re-standardized version Questionnaire (5-15R). 5-15R, a re-standardized version of Five to Fifteen Questionnaire, assessing neurodevelopmental functioning in children (Korkman et al., 2004). In the present study, parents completed subscales assessing Learning Skills (reading, writing, and mathematics), Language, General Learning Difficulties, Exceptional Skills, and Artistic/Practical Skills. Items are rated on a 3-point scale (0 = does not apply, 1 = applies sometimes or to some extent, 2 = definitely applies), with higher scores indicating greater difficulty. This questionnaire has shown strong psychometric properties, including acceptable to good internal consistency, test–retest reliability, and inter-rater agreement (Korkman et al., 2004).

#### 2.4.2. Emotion and Cognitive Screening (Session 1)

Wechsler Abbreviated Scale of Intelligence—Second Edition (WASI-II). This is a standardized intelligence test used to estimate intelligence (Irby & Floyd, 2013). In the current study, this was included as a brief screening measure to ensure the child’s cognitive ability was within the average range, and was not used for diagnostic purposes. A two-subtest version was administered remotely to assess participants’ general cognitive ability. The Vocabulary subtest assessed verbal knowledge and expressive language, and the Matrix Reasoning subtest assessed nonverbal fluid reasoning. Age-normed standard scores from these two subtests were combined to estimate full-scale IQ. The WASI-II has demonstrated strong reliability and validity for estimating cognitive functioning in both clinical and non-clinical populations.

Cambridge Mindreading Face-Voice Battery for Children (CAM-C). This task was used to assess complex emotion recognition in children with ASD (Golan et al., 2015). The task consisted of two subtasks: (1) Face Subtask—participants watched silent video clips of actors displaying emotional expressions; and (2) Voice Subtask—participants listened to audio recordings of emotional speech without visual cues. Nine emotions were assessed (i.e., amused, bothered, disappointed, embarrassed, jealous, loving, nervous, undecided, and unfriendly). Each emotion was presented in six trials (three per subtask), yielding a total of 54 trials. On each trial, participants selected the emotion label that best matched the stimulus from four response options. Responses were scored as correct or incorrect (1 or 0), and total accuracy scores were calculated separately for the Face (maximum = 27) and Voice (maximum = 27) conditions, with higher scores indicating better emotion recognition performance. The Voice subtask was introduced later in the study, resulting in a smaller number of participants completing this subtask.

#### 2.4.3. Visual and Cognitive Tasks (Session 2)

Orientation Discrimination (OD) Task. This task evaluated participants’ ability to discriminate fine spatial orientation differences (Skottun et al., 1987). Performance was measured through a two-alternative forced-choice (2AFC) staircase procedure. Participants completed six practice trials prior to main task to familiarize themselves with the task format. Each trial started with a fixation cross, followed by a Gabor patch presenting 128 millisecond (ms), tilted either clockwise or counterclockwise relative to vertical (see Figure 2a). Participants indicated the direction of tilt on each trial. Task difficulty was modulated by a 3-down-1-up (i.e., 3 consecutively correct responses make the task harder, with any incorrect responses the task becomes 1 step easier) adaptive staircase (converging at 79.4% accuracy). The main task started at an orientation difference of 45° and decreased in logarithmic steps to a minimum of 0.72°. The task ended after five reversals or 60 trials. The threshold was defined as the orientation delta (°) of the final trial, with lower values indicating higher orientation sensitivity.

Contour Integration (CI) Task. This task evaluated participants’ ability to perceive global structure by detecting shapes embedded in visual noise (Field et al., 1993). Each trial displayed approximately 440 Gabors on the screen, with 16 aligned Gabors forming a circular contour (see Figure 2b). Participants completed seven practice trials prior to the main task to ensure task comprehension. On each trial, participants had 1500 ms to tap anywhere within the perceived contour. Task difficulty was manipulated by the orientation jitter (i.e., angular deviation from perfect alignment), bounded between 0° and 90°. A hybrid staircase was used: a standard 3-down-1-up rule with a “streaking” feature that temporarily switched to a 1-up rule after four consecutive correct responses to accelerate difficulty. The task terminated after five reversals or 60 trials. The threshold was defined as the jitter level (°) of the last completed trial, with higher values indicating greater sensitivity to global visual structure.

Corsi Task. This task evaluated visuospatial working memory capacity with two subtasks: forward and backward span (Kessels et al., 2000). Each subtask began with two practice trials. On each trial, cartoon gophers appeared sequentially at different screen locations (see Figure 2d). In the forward span subtask, participants reproduced the sequence in the same order; in the backward span subtask, they reproduced the sequence in reverse order. The task began with a sequence length of two and increased by one following a correct response. If a response was incorrect, a second trial of the same sequence length was presented. Two consecutive incorrect responses resulted in a decrease of two sequence units and the loss of one life. Participants had two lives in total, and the task terminated after two sequence levels were failed twice consecutively. Sequence length never dropped below two. The primary outcome measure was the longest forward or backward sequence length correctly recalled at least once.

N-Back Task. This task evaluated working memory updating using a fixed 1-back paradigm (Kirchner, 1958). Participants viewed a sequence of animal images and were prompted to tap the screen when the current image matched with the one shown immediately before (see Figure 2c). Ten practice trials were administered prior to the task, followed by 61 test trials. Performance was quantified as accuracy, with higher scores indicating better working memory performance.

UCancellation Task. This task evaluated inhibitory control (Pahor et al., 2022). Participants completed two practice trials prior to the main task. On each trial, participants were shown a row of eight cartoon monkeys that varied in orientation (upright or upside-down), facing direction (left or right), and color (light-brown or dark-brown). The target stimulus was a dark-brown monkey that was upside-down and facing left (see Figure 2e). Participants were instructed to tap only target stimuli while scanning from left to right. Each row displayed presented for up to 6 s, and participants completed as many rows as possible within a 3 min time limit. Performance was quantified as concentrated performance, calculated by subtracting total false alarms from total correct hits. Higher scores indicated better inhibitory control.

#### 2.4.4. Auditory Tasks (Session 3)

Pure Tone Task (PTT). This task evaluated minimum audibility under two subtasks: quiet and noisy (American National Standards Institute, 2004; Lelo de Larrea-Mancera et al., 2020). Participants were asked to detect a 2000 hertz (Hz) pure tone presented either in silence (quiet subtask) or embedded in continuous broadband white noise at 60 dB SPL (noise subtask). On each trial, stimulus was presented for 500 ms, followed by a 2AFC response screen on which participants indicated whether they heard the tone (see Figure 2f). In the quiet subtask, tone intensity followed a fixed series (70, 50, 40, 30, 20, 10, 5, and 0 dB SPL). In the noise condition, tone intensity decreased in 5 dB steps every three trials, starting at 70 dB SPL and continuing to a minimum of 10 dB SPL. Catch trials (0–2 per block of six) were included to reduce bias. The task ended if the participant either produced four consecutive incorrect responses or reached the lowest tone level. The threshold was defined as the final correct intensity level, with lower thresholds reflecting better auditory sensitivity.

Gap in Noise (GIN) Task. This task assessed temporal sensitivity, defined as the ability to detect brief silent gaps in continuous noise (Gallun et al., 2014; Lelo de Larrea-Mancera et al., 2020). Participants were instructed to detect brief silent gaps in white noise bursts. Each trial consisted of four auditory intervals, represented on screen by four gray squares that changed color (blue) during playback (see Figure 2g). Each interval contained two 4 ms white noise bursts (cropped Gaussian), presented diotically at 70 dB SPL. A silent gap was inserted into one of the two middle intervals. Participants selected the interval containing the gap, and no feedback was provided following responses. Task difficulty was adjusted using a two-stage adaptive staircase procedure with a 2-down–1-up rule. In Stage 1, gap duration began with a 20 ms gap and 5 ms steps, ending after three reversals. In stage 2 gap duration was adjusted in 2 ms steps and ended after six additional reversals. The final gap detection threshold was computed as the mean of the last six reversals in Stage 2. Gap durations ranged from 0 to 40 ms, with lower thresholds reflecting better temporal resolution.

Digits in Noise (DIN) Task. This task assessed general speech-in-noise recognition ability (Lelo de Larrea-Mancera et al., 2020; Smits et al., 2013). Participants needed to identify spoken digit triplets (0–9) embedded in continuous broadband white noise presented diotically at 65 dB SPL. On each trial, a digit triplet was presented with 500 ms interstimulus intervals between digits, as well as 500 ms of silence padding at stimulus onset and offset. Participants responded via an on-screen keypad and received visual feedback after each trial (see Figure 2h). Task difficulty was adjusted using a 1-down-1-up staircase procedure based on target-to-masker ratio (TMR), starting at a 0 dB with 2 dB step sizes and bounding between −16 dB and +10 dB. Thresholds were calculated by averaging the last six reversals per run, and the final TMR threshold was the mean across two 25-trial runs. Lower TMR values indicated better speech-in-noise perception.

Spatial Release Masking (SRM) Task. This task assessed participants’ ability to use spatial cues to distinguish target speech from competing talkers (Lelo de Larrea-Mancera et al., 2020; Marrone et al., 2008). On each trial, participants listened to a target sentence (“Charlie goes to the [color] [number]”) drawn from the Coordinate Response Measure (CRM) corpus (Bolia et al., 2000) and identified the perceived color-number pair using an on-screen grid (see Figure 2i). The task comprised three subtasks. In the single talker subtask, only the target sentence was presented. In the co-located subtask, the target was presented simultaneously with two masking sentences from different talkers, all originating from 0° azimuth. In the separated subtask, the target remained at 0°, while the maskers were spatially positioned at ±45°, on the azimuth, using head-related transfer functions (HRTFs). Task difficulty in the single talker subtask was adjusted using a 1-up–1-down adaptive staircase procedure with 5 dB step sizes over 20 trials. Thresholds were calculated as the average level across the final six reversals. In the colocated and separated subtasks, masker levels increased linearly from 55 to 75 dB SPL in 2 dB steps every two trials, while the target level remained fixed. Masker thresholds were estimated at the level corresponding to approximately 50% accuracy. TMR thresholds were calculated by subtracting the masker level from the fixed target level, with lower TMRs indicating better speech-in-noise perception and greater spatial release from masking.

Spectrotemporal Modulation (STM) Task. This task assessed participants’ sensitivity to dynamic changes in both frequency and time, which are critical for decoding complex auditory signals such as speech (Bernstein et al., 2013; Stavropoulos et al., 2021). Participants were presented with four sequential noise carriers (300 ms each, centered at 2000 Hz) at 70 dB SPL (same format with Figure 2g). The task included two subtasks. In the detection subtask, one interval contained a spectrotemporally modulated signal (2 cycles/octave; 3.33 Hz), whereas the remaining intervals were unmodulated. In the discrimination subtask, all intervals were spectrotemporally modulated, but one interval differed in modulation direction (upward vs. downward). Participants identified the target interval by selecting the corresponding option on an on-screen response grid. Task difficulty was controlled by adjusting modulation depth using a two-stage 2-up-1-down adaptive staircase: Stage 1 used 0.5 dB steps for three reversals, followed by 0.2 dB steps in Stage 2 until nine total reversals were reached. The final threshold was calculated as the average of the last six reversals in Stage 2, with lower threshold indicating better spectrotemporal sensitivity.

### 2.5. Data Quality Coding

#### 2.5.1. Coding Procedures

To evaluate data quality and task feasibility, two examiners independently reviewed each participant’s trial-by-trial performance alongside session notes. Based on this review, a detailed coding scheme was developed for all tasks, outlining specific criteria for data inclusion and exclusion. Using the finalized code scheme, one examiner initially coded all completed tasks for each participant. For tasks that included multiple subtasks (e.g., Corsi Forward and Corsi Backwards are different subtasks within the Corsi Block task), each subtask was coded separately to account for cases in which a participant was unable to complete one subtask but successfully completed another. To assess coding consistency, a second rater independently coded a randomly selected 10% subset of the data. Agreement between raters was 100% for this subset. In addition, all cases identified by the primary rater as requiring exclusion were reviewed by the second rater. Data was excluded from a specific subtask only if both examiners agreed on their exclusion status.

#### 2.5.2. Code Scheme

Participants’ performance in each task or subtask was classified into one of the following categories to determine data usability.

##### Inclusion Categories

This includes two categories.

Consistent Performance. Participants were classified as showing consistent performance if they completed the task as intended and demonstrated response patterns consistent with task instructions. Performance trajectories followed expected trends (i.e., thresholds converging appropriately where the participant was responding consistently enough for a reliable threshold to be estimated), and no technical disruptions were reported. Data meeting these criteria were included in the final analyses.

Inconsistent Performance. Participants were classified as showing poor compliance if they demonstrated reduced engagement or attentional difficulties during the task—such as a sudden and sustained increase in errors after a certain trial point. In these cases, only task segments that reflected expected performance were retained, and outcome measures (e.g., thresholds or accuracy scores) were recalculated based on the usable portion of the data. This partial-data retention approach was adopted to preserve valid information while minimizing the influence of disengagement on data quality.

##### Exclusion Categories

This includes three categories.

Unable to Complete. Participants were classified as unable to complete a task if they were unable to engage meaningfully, resulting in uninterpretable or invalid data. This included cases where participants consistently responded incorrectly or failed to stabilize at a meaningful threshold, suggesting a lack of perceptual sensitivity or task comprehension. For example, in the Pure Tone Detection task, some participants responded “Yes” on every trial, including catch trials, indicating a response bias rather than true detection. In other instances, RAs observed behaviors such as random clicking or clear signs of inattention despite reminders, reflecting disengagement. These patterns indicated that the task could not be completed in a valid manner.

Technical Issues. Participants were classified as having technical issues if their session was affected by technological problems that interfered with stimulus presentation or response recording. These issues included audio playback failure, broken headphones, frozen screens, or application crashes, all of which compromised data integrity.

Background Noise. Participants were classified under background noise if excessive environmental noise (e.g., conversation, television, traffic) was noted by the RA during the session. Such noise could mask auditory stimuli or distract participants, particularly in tasks requiring fine acoustic discrimination (e.g., Digits-in-Noise or Gap-in-Noise). Data from these sessions were flagged for potential exclusion depending on task sensitivity.

### 2.6. Data Analysis

Data analysis was conducted in two sequential phases. The first phase focused on characterizing feasibility and data usability across tasks using the predefined data quality coding scheme. For each task and subtask, the proportion of participants classified as producing usable data (i.e., Consistent Performance or Inconsistent Performance with usable segments retained) was calculated. Exclusion frequencies were summarized by category (Unable to Complete, Technical Issues, Background Noise) to identify common sources of data loss across the assessment battery. To examine whether exclusion frequencies differed across tasks or subtasks, chi-square tests of independence were conducted on exclusion classifications. Chi-square analyses were based on subtask-level coding outcomes and used two-tailed tests with an alpha level of 0.05.

The second phase of analysis focused on descriptive characterization of task performance among participants whose data met inclusion criteria. For each task and subtask, performance was summarized using descriptive statistics. To visualize the distribution, variability, and central tendency of performance across participants, violin plots were generated for each task.

## 3. Results

### 3.1. Feasibility and Data Usability Across Tasks

Across the full assessment battery, most participants produced usable data with an average of 85% of participants per task met inclusion criteria for analysis. As shown in Table 2, subtask-level inclusion rates varied across individual measures, with inclusion rates remaining high across tasks spanning cognitive, visual, and auditory domains. Results indicated that the majority of tasks could be completed successfully under remote, supervised testing conditions.

To characterize sources of data loss, exclusion frequencies were also summarized across tasks (see Table 2). The most common reasons for exclusion were participants being unable to complete a task meaningfully, followed by technical issues and background noise. Importantly, exclusion patterns were broadly distributed across tasks rather than concentrated in any single measure. Chi-square tests showed that there was no significant association between task type and exclusion classification (χ^2^ = 74.59, *p* = 0.996), indicating that task failures were not systematically driven by a particular task.

### 3.2. Descriptive Task Performance

An overview of descriptive performance outcomes across the full assessment battery is provided in Table 2, which summarizes task-level central tendency, variability, and expected performance ranges. For cognitive and visual measures, results are reported on a task-by-task, while auditory performance is summarized by functional domain. Across tasks, observed performance values largely fell within ranges reported in prior studies or expected based on task specifications, supporting the interpretability of remotely collected data.

#### 3.2.1. Session 2 Tasks

##### Cognitive Processing

**Figure 3:**
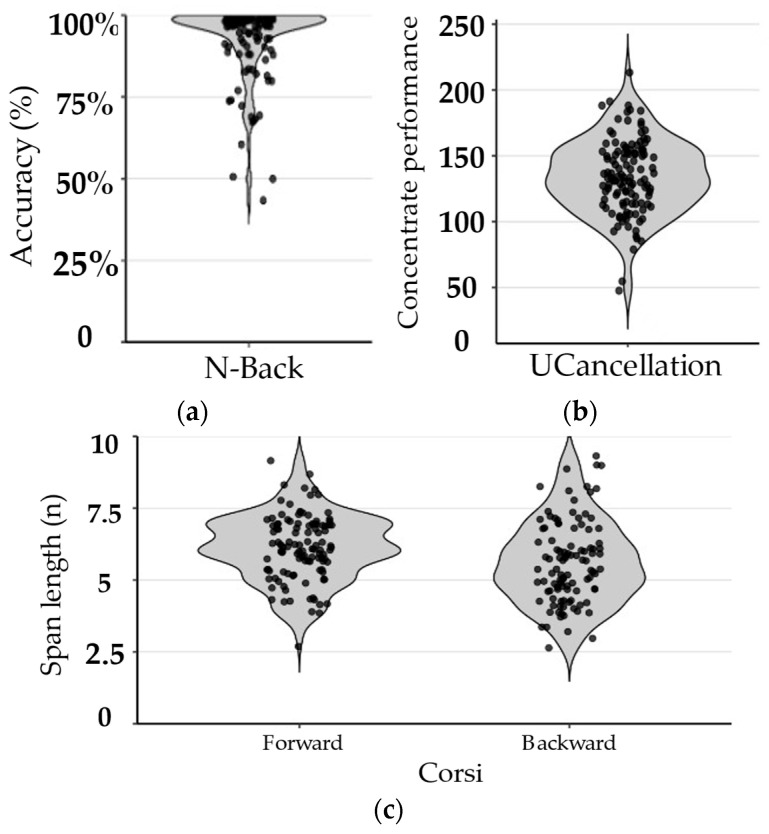
Cognitive Task Performance. Violin plots illustrate the distribution, density, and individual data for performance on cognitive tasks among participants whose data met inclusion criteria. Panels are listed as: (**a**) accuracy for the N-Back task; (**b**) concentrated performance scores for the UCancellation task; (**c**) span length for the Simple Corsi task under forward and backward subtasks. Each point represents an individual participant.

For the cognitive tasks we found that most participants showed usable data with overall performance falling within expected ranges for these tasks (see Figure 3). For the n-back, 93% of the participants showed usable data and with overall high accuracy (93 ± 12%). For the Span tasks, the forward span showed 90% of participants with consistent performance and 5% of participants with inconsistent performance with overall span scores of (6.1 ± 1.2). There were fewer participants showing consistent performance (79%) with 9% showing inconsistent performance and 12% unable to complete the tasks; however, for those who did complete, performance (5.6 ± 1.4) fell within the normal range (e.g., (Farrell Pagulayan et al., 2006; Wilde & Strauss, 2002)). For the UCancellation task, 100% of participants showed consistent performance with concentration performance scores falling within the normal range for this task (134 ± 28) (Pahor et al., 2022). These data indicate stable and interpretable task performance for cognitive tasks under remote administration where most participants could perform the tasks well, although with some participants struggling on the backward corsi task compared to the others.

##### Visual Processing

**Figure 4:**
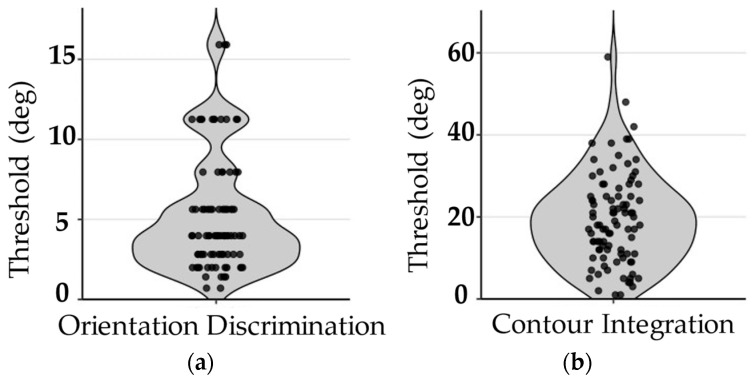
Visual Task Performance. Violin plots illustrate the distribution, density, and individual data for performance on cognitive tasks among participants whose data met inclusion criteria. Panels are listed as: (**a**) angular threshold for the orientation discrimination task; (**b**) jitter threshold for the contour integration task. Each point represents an individual participant.

For the visual tasks, a majority of participants produced usable data, with overall performance falling within expected ranges (see Figure 4). For orientation discrimination, 69% of participants showed consistent performance and 3% showed inconsistent performance, with angular difference thresholds (5.2° ± 3.6) being within the typical range reported in prior studies (Lewis et al., 2007). For contour integration, 66% of participants demonstrated consistent performance and 10% showed inconsistent performance, with jitter thresholds (19.3° ± 11.0) generally consistent with previously published values (Jayakumar et al., 2024). These findings indicate that visual perceptual thresholds could be reliably estimated under supervised remote administration, despite modestly lower usable data rates compared to cognitive tasks.

#### 3.2.2. Session 3 Tasks

##### Minimum Hearing Ability

**Figure 5:**
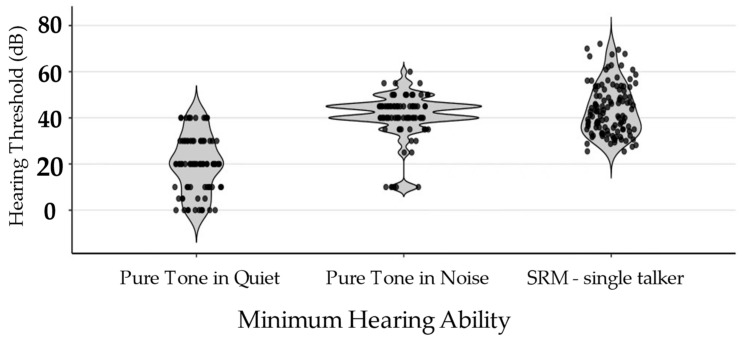
Minimum Hearing Ability Task Performance. Violin plots illustrate the distribution, density, and individual data for performance on cognitive tasks among participants whose data met inclusion criteria. Each point represents an individual participant.

For minimum hearing ability, we also have a majority of participants producing usable data across subtasks, with overall thresholds falling within interpretable ranges (see Figure 5). For pure tone detection in quiet, 71% of participants produced usable data, with average thresholds of 20.06 ± 12.50 dB. These values are slightly elevated relative to typical normal-hearing thresholds (<27 dB SPL at 2000 Hz; (World Health Organization, 2021)). For pure tone detection in noise, 74% had usable data, with thresholds of 40.80 ± 9.71 dB. For the SRM single-talker subtask, 98% showed consistent performance, with speech detection thresholds of 43.54 ± 10.97 dB. This is higher than prior work using the same remote subtask, reporting average thresholds of approximately 32 dB SPL in healthy undergraduates (Gallun et al., 2018). These findings indicate that auditory threshold estimates were interpretable under remote administration.

##### Central Auditory Processes

**Figure 6:**
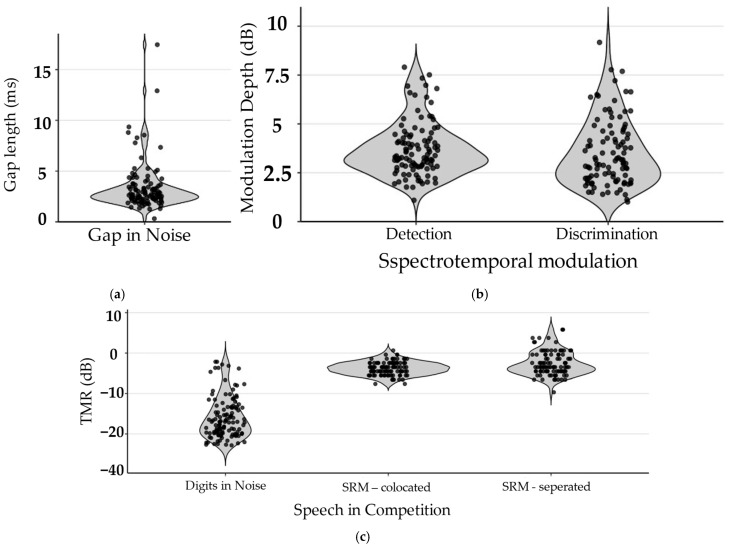
Central Auditory Task Performance. Violin plots illustrate the distribution, density, and individual data for performance on cognitive tasks among participants whose data met inclusion criteria. Panels are listed as: (**a**) threshold for the gap in noise task; (**b**) depth threshold for the spectrotemporal modulation task; (**c**) speech-in-competition performance expressed as target-to-masker ratio (TMR) for digits in noise and spatial release from masking tasks. Each point represents an individual participant.

In the central auditory processing tasks, a majority of participants produced usable data, with overall performance falling within expected ranges (see Figure 6). For gap in noise (GIN), 88% of participants showed consistent performance and 0.84% showed inconsistent performance, with gap length thresholds of 3.39 ± 2.33 ms. Speech-in-competition ability was evaluated using digits in noise (DIN) and two spatial release from masking (SRM) subtasks (colocated and separated). For DIN, 95% of participants showed consistent performance and 5% showed inconsistent performance, with thresholds of −15.98 ± 5.1 dB TMR, within the expected range of approximately −23 to −16 dB TMR (Coppola et al., 2026). For the SRM colocated subtask, 79% showed consistent performance and 9% showed inconsistent performance, with thresholds of −3.77 ± 1.64 dB TMR, consistent with reported values (Lelo de Larrea-Mancera et al., 2020). In the separated subtask, 81% showed consistent performance and 4% showed inconsistent performance, with thresholds of −2.60 ± 2.93 dB TMR, also within the anticipated range for spatial separation effects (Lelo de Larrea-Mancera et al., 2020). Spectrotemporal modulation sensitivity was assessed using modulation depth thresholds in detection and discrimination subtasks. For STM detection, 82.61% of participants showed consistent performance and 7.76% showed inconsistent performance, with modulation depth thresholds of 3.73 ± 1.39 dB, within expected ranges reported for comparable paradigms (Lelo de Larrea-Mancera et al., 2020). For STM discrimination, 72.72% showed consistent performance and 8.62% showed inconsistent performance, with thresholds of 3.53 ± 1.70 dB. These findings indicate that auditory perceptual thresholds could be reliably estimated under supervised remote administration.

## 4. Discussion

The current results support the feasibility of delivering a comprehensive, sensory–cognitive remote assessment battery in a sample of children with ASD. Our results indicate high inclusion rates across most tasks, minimal technical exclusions, and generally interpretable performance distributions. These findings are particularly encouraging given the length and complexity of the battery, and the challenges typically associated with remote testing in pediatric and neurodiverse populations.

Feasibility of remote assessment varied across domains, reflecting differences in task order, design feature, environment conditions, and stimuli algorithm. Cognitive tasks demonstrated the highest feasibility, with approximately 94% of participants producing usable data, followed by auditory tasks (87%) and visual tasks (75%). One likely contributor to this pattern is task order (Charness et al., 2012). Visual tasks were administered first within the BGCScience, and lower usability may reflect participants’ initial unfamiliarity with both the software interface and psychophysical task demands. By contrast, cognitive tasks were administered after the visual tasks, at a point when participants were already accustomed to the interface and response structure, potentially reducing operational errors and misunderstandings.

These observations suggest actionable improvements for future implementations. While the cognitive tasks had elaborated visual tutorials the demonstrated how to conduct the tasks, the hearing and vision tasks only had written instructions, which some participants may skip or not fully process. The difference in the number of participants producing usable data may reflect a need to clarify the instructions. To this end, we are actively developing revised tutorials that minimize text and instead rely on images and animations to communicate task rules in a more accessible and engaging format. Further, increasing the number of practice trials and having RAs explicitly confirm rule com-prehension prior to initiating the main task may further improve usability (Crump et al., 2013).

In addition to task order, design features may also have contributed to the observed differences. All cognitive tasks were gamified, which may have supported sustained engagement. Prior work suggests that game-based paradigms can enhance motivation in children with ASD (Grynszpan et al., 2014), and this design element may have contributed to the particularly high usability observed for executive function measures.

Auditory tasks, while still demonstrating strong feasibility overall, showed lower usability relative to cognitive measures. This pattern is consistent with the inherent sensitivity of auditory testing to environmental conditions (Woods et al., 2017). Despite explicit instructions to maintain a quiet environment, RA frequently documented background noise that caregivers were unable to fully control in home settings. Moreover, the adaptive algorithm used in all auditory tasks began with relatively high stimulus intensities as part of a descending staircase. Some participants reported that these initial stimuli were uncomfortably loud, which negatively impacted engagement. Although this issue had not emerged in prior implementations with typically developing samples, individuals with ASD may exhibit heightened sensory sensitivity (Khalfa et al., 2004), such that intensities tolerable for neurotypical listeners may be distressing. These findings suggest that future remote auditory protocols for ASD populations may benefit from initiating tasks at intermediate stimulus levels rather than the highest intensities, thereby reducing discomfort while preserving measurement validity.

Beyond feasibility, descriptive performance patterns provide additional insight. Across most tasks, mean performance fell within expected ranges reported for comparable paradigms, suggesting that remotely administered adaptive procedures yielded valid and interpretable estimates of perceptual and cognitive functioning. An exception was observed in the minimum hearing measures, where thresholds were higher relative to reference values obtained using the same remote paradigms in prior samples. Importantly, methodological explanations are unlikely to fully account for this pattern. The comparison data were derived from identical task implementations administered remotely within the same laboratory framework, reducing concerns regarding paradigm or platform differences. In addition, participants who failed to demonstrate task understanding were excluded from analyses, making systematic misunderstanding an unlikely primary driver of elevated thresholds. One possibility is that the upward shift in mean thresholds—and the relatively large variability observed—reflects genuine heterogeneity in low-level auditory sensitivity within the ASD population (O’Connor, 2012). That is, a subset of participants may exhibit subtle peripheral or low-level auditory processing differences. While the present study was not designed to diagnose hearing impairment, this distributional pattern is consistent with the broader premise motivating this work: that variability in basic perceptual processing may underlie meaningful differences in higher-level functioning. Identifying such low-level sensory variability may ultimately inform more individualized approaches to intervention, particularly if specific perceptual profiles are linked to downstream communication outcomes.

Several limitations should be considered when interpreting the findings of the present study. First, ASD diagnoses and certain participant characteristics (e.g., vision status) were based on parent report and were not independently verified through clinical evaluation. In addition, the sample was relatively high-functioning and selected based on specific inclusion criteria (e.g., IQ ≥ 70, absence of reported comorbidities, and access to compatible technology). This limits the availability of objective clinical information and may restrict generalizability to the broader ASD population. Second, the analyses conducted in this study were descriptive statistics, focusing on data usability and performance distributions rather than inferential comparisons. Therefore, conclusions are limited to feasibility and interpretability of task performance under remote testing conditions. Third, the protocol employed a fixed task order, which may have introduced learning or fatigue effects across sessions. As discussed above, task order may have influenced feasibility outcomes, particularly for early visual tasks and later auditory tasks. The fourth limitation is the relatively large number of tasks administered across sessions, which may have increased participant burden and introduced variability in engagement or fatigue, particularly in a remote testing context. Future implementations may benefit from counterbalancing task order or introducing adaptive sequencing to further optimize feasibility across domains. Finally, although the tasks were derived from well-established paradigms, their psychometric properties have not been formally validated in the present remote, app-based implementation. Factors inherent to remote testing, including variability in device characteristics, environmental conditions, and the use of gamified interfaces, may influence performance in ways that differ from traditional laboratory settings. Accordingly, the current findings should be interpreted as evidence of feasibility and interpretability rather than formal validation of these measures. Future work should directly evaluate the reliability and validity of these tasks under remote conditions, including comparisons with in-lab assessments and examination of test–retest reliability.

The present findings have several methodological implications for the design and implementation of remote sensory–cognitive assessments in ASD research. First, the results highlight the importance of structured supervision in remote settings. Although assessments were conducted outside the laboratory, real-time support from trained RA played a critical role in ensuring task comprehension, maintaining engagement, and documenting contextual factors. These findings suggest that supervised remote protocols may offer a practical balance between scalability and data integrity, particularly for pediatric ASD populations. Second, task design features appear to meaningfully influence feasibility under remote conditions. Gamified cognitive tasks demonstrated high data usability, consistent with prior evidence that game-based paradigms can support motivation and sustained engagement in children with ASD. In contrast, tasks administered early in the protocol or those requiring fine perceptual judgments were more sensitive to initial unfamiliarity with software and task demands. Together, these observations underscore the value of intuitive interface design, streamlined tutorials, and sufficient practice trials to support successful remote task execution. Third, auditory measures, while feasible, were particularly sensitive to environmental noise and individual differences in sensory sensitivity. These results suggest that remote auditory assessments may benefit from adaptive stimulus calibration, more flexible starting levels, or enhanced environmental screening procedures to improve participant comfort and data usability without compromising interpretability. Finally, the present study demonstrates the feasibility of integrating multiple sensory and cognitive measures within a single remote protocol. Such multi-domain batteries offer a promising avenue for capturing individual variability across sensory and cognitive processes in ASD, while reducing barriers associated with in-lab testing.

## 5. Conclusions

This study provides preliminary but compelling evidence that remote assessments are feasible for children with autism. High inclusion rates and interpretable performance patterns suggest that meaningful behavioral data can be collected outside traditional laboratory settings. Such tools hold promises not only for future large-scale online research, but also for personalized assessment and intervention.

## Figures and Tables

**Table 1:** Demographics Characteristics of Participants.

Variables	Participants (N = 121)
Mean age in years (SD) Range	11.31 (1.38) 8–14
Gender (Male/Female/NB)	93/27/1
Mean SRS T-score (SD) Range	73.85 (10.74) 44–90
Mean 5–15 scores (SD) Range	13.43 (7.99) 0–34
Mean WASI FSIQ scores (SD) Range	108.29 (17.75) 71–145

**Table 2:** Task-Level Data Usability and Descriptive Performance Across the Remote Assessment Battery.

Task	Performance Category					N	Mean	SD
	Inclusion		Exclusion					
	Consistent Performance	Inconsistent Performance	Unable to Complete	Technical Issue	Background Noise			
N-back	112 (92.56%)		9 (7.35%)			112	93%	12%
Simple Corsi Forward	107 (89.92%)	6 (5.04%)	6 (5.04%)			113	6.11	1.15
Simple Corsi Backward	94 (78.99%)	11 (9.24%)	14 (11.76%)			105	5.61	1.42
UCancellation	120 (100%)					120	134.56	28.27
Orientation Discrimination	84 (69.42%)	4 (3.31%)	33 (27.27%)			88	5.24°	3.61
Contour Integration	81 (66.94%)	12 (9.92%)	22 (18.18%)	6 (4.96%)		93	19.36°	11.07
Puretone in Quiet	85 (71.43%)		32 (26.89%)	1 (0.84%)	1 (0.84%)	85	20.06 dB	12.5
Puretone in Noise	88 (73.95%)		30 (25.21%)	1 (0.84%)		88	40.8 dB	9.71
Gap in Noise	105 (88.24%)	1 (0.84%)	12 (10.08%)		1 (0.84%)	106	3.39 ms	2.33
Digit in Noise	113 (94.96%)	6 (5.04%)				119	−16.09 dB	4.96
SRM-single talker	116 (98.31%)		2 (1.69%)			116	43.54 dB	10.97
SRM colocated	93 (78.81%)	11 (9.33%)	14 (11.86%)			104	−2.60 dB	2.93
SRM seperated	95 (81.20%)	5 (4.27%)	17 (14.53%)			100	−3.77 dB	1.64
STM detection	95 (82.61%)	9 (7.76%)	11 (9.48%)			104	4.11 dB	1.86
STM discrimination	89 (76.72%)	10 (8.62%)	17 (14.65%)			99	3.52 dB	1.69

Note. Each cell in the Inclusion and Exclusion columns reports the number of participants classified in that category, followed by the corresponding percentage of the total sample for that task. N usable reflects the number of participants whose data met inclusion criteria (Consistent Performance or Inconsistent Performance with usable segments retained) and were included in performance analyses. Mean values are reported in task-specific units.

## Data Availability

Dataset available on request from the authors.

## Abbreviations

The following abbreviations are used in this manuscript:

ASD	Autism Spectrum Disorder
RA	Research assistant
SPARK	Simon Powering Autism Research
SRS-2	Social Responsiveness Scale–Second Edition
5-15 R	Five to Fifteen re-standardized version Questionnaire
WASI-II	Wechsler Abbreviated Scale of Intelligence—Second Edition
CAM-C	Cambridge Mindreading Face-Voice Battery for Children
OD	Orientation Discrimination
CI	Contour Integration
PTT	Pure Tone Task
GIN	Gap in Noise
DIN	Digits in Noise
SRM	Spatial Release Masking
TMR	target-to-masker ratio
STM	Spectrotemporal Modulation
